# Leprosy ulcers in a rural hospital of Ethiopia: pattern of aerobic bacterial isolates and drug sensitivities

**DOI:** 10.1186/s12941-014-0047-z

**Published:** 2014-09-17

**Authors:** José M Ramos, Ramón Pérez-Tanoira, Cristina García-García, Laura Prieto-Pérez, María C Bellón, Fernando Mateos, Gabre Tisisano, Tafese Yohannes, Francisco Reyes, Miguel Górgolas

**Affiliations:** Department of Medicine and Laboratory, Gambo Rural General Hospital, Kore, West-Arsi, Gambo, Ethiopia; Department of Internal Medicine, Hospital General Universitario de Alicante, Alicante, Spain; Department of Medicine, Universidad Miguel Hernández, San Juan Campus, C/ Pintor Baeza s/n, 03010 Alicante, Spain; Department of Clinical Microbiology, Fundación Jiménez Díaz, Madrid, Spain; Division of Infectious Diseases, Fundación Jiménez Díaz, Madrid, Spain; Department of Internal Medicine, Hospital General Virgen de la Luz, Cuenca, Spain; Departament of Medicine, Universidad Autónoma de Madrid, Madrid, Spain; Department of Internal Medicine, Hospital General Universitario de Albacete, Albacete, Spain

**Keywords:** Bacterial isolation, Chronic ulcer, Leprosy, Pathogens, Susceptibility testing

## Abstract

**Background:**

Plantar ulcers, which commonly occur in leprosy patients, tend to recur increasing physical disability. The aim of this study is to identify both the bacteriological profile of these ulcers and the antibiotic susceptibility of the isolated bacteria.

**Materials and methods:**

68 leprosy patients with chronic ulcers attending the in-patient department of Gambo General Hospital, West Arsi, were included in this study. Proper sample collection, inoculation on culture media, and final identification using biochemical methods were undertaken.

**Results:**

66 patients (97.1%) had a positive culture. A total of 81 microorganisms were isolated. Multiple organisms (two or more) were isolated in 15 (22.7% out of positive culture) patients. The main isolation was *Proteus* spp (30.9%), followed by *Escherichia coli* (21.0%), *Staphylococcus aureus* (18.5%) and *Pseudomonas aeruginosa* (9.9%). In the total number of the isolated bacteria, the antibiotics with less resistance were gentamicin (18.5%), fosfomycin (22.2%) cefoxitin (24.7%), ceftriaxone (25.9%) ciprofloxacin (25.9%), and amoxicillin-clavulanic acid (28.49%).

**Conclusion:**

The bacteriological study of plantar ulcers of leprosy patients revealed Enterobacteriaceae and *S.* aureus as the main pathogens involved in such infections. The results of this study may guide empirical therapy in a rural area hospital where culture and susceptibility testing facilities are scarce.

## Background

Leprosy is a chronic infectious disease caused by the obligate intracellular pathogen *Mycobacterium leprae* [1], and still remains a public health problem, mainly in Africa, Asia and Latin America [2]. It has many complications including: leprosy reactions, development of plantar and palmar ulcerations, lagophthalmos (loss of eyelid function) and corneal anesthesia [3]. Chronic ulcers are included among the most serious complications of leprosy; these are highly infected with bacteria, which delays the healing process [4], and furthermore, they usually recur, which in such cases increase the physical disability [4]. There is little information about the pattern of bacterial isolates and drug sensitivities of infected ulcers in leprosy patients with leprosy, and most studies have been carried out in India [4-9]. Some studies have been performed in Africa [10,11], but to our knowledge only three have been made in Ethiopia [12-14]. Two of these studies were carried out in 1970 and 1989, and the third in 2006; they assessed the bacteriology of infected ulcers and the sensitivity of these organisms to available antimicrobials in leprosy patients who visited the ALERT hospital, and the ALERT Hospital, Kuyera Hospital and Gambo General Hospital (GGH) [12-14]. In Ethiopia there are four main hospitals providing special care for leprosy patients: ALERT Hospital, Kuyera Hospital, Bisidimo Hospital and GGH. GGH is a rural center with some laboratory facilities, but it is not provided with any procedure for the isolation and identification of bacteria and drug sensitivities.

This is a descriptive cross-sectional prospective study which aims was to identify the isolates from infected ulcers and drug sensitivities of microorganism isolation over a period of 4 months.

## Material and methods

### Setting

GGH is a rural hospital in Ethiopia, which is a referral institution in the leprosy care program in the country according to the guidelines of the Tuberculosis and Leprosy Prevention and Control Programme (TLPCP) of the Ministry of Health of Ethiopia. The GGH is located in the West-Arsi zone, 250 km south of Addis Ababa.

### Type of study

A descriptive cross-sectional prospective study from July 2013 to December 2013.

### Case selection

Patients admitted to GGH with ulcers that were chronic, indolent, with scanty discharge and a pale, unhealthy fibrosis base were included. Diagnosis of osteomyelitis was based on the clinical assessment and physical exam along with radiographic examinations.

### Sample collection

Samples were taken from pus produced by either the ulcer or from the depth of the ulcer with a sterile bacteriological loop.

### Identification

Swabs were processed for gram stain and culture. For isolation of the aerobes, inoculation was done on nutrient agar, blood agar, MacConkey’s agar and Mannitol agar media and incubated overnight at 37°C, and also Sabouraud agar at room temperature. Identification of the isolates was performed using biochemical methods [15] in cases where there was some doubt about identification. The microorganisms were identified by Matrix-Assisted Laser Desorption/Ionization Time-Of-Flight (MALDI-TOF) in Spain [16,17]. The isolates were further tested for antibiotic sensitivity to different classes of antimicrobials on Mueller Hinton agar medium, using Kirby-Bauer disc diffusion method and following the guidelines of the Clinical and Laboratory Standards Institute [18]: (1) Cephalosporin class (cefoxitin, ceftriaxone); (2) Aminoglycosides class (gentamycin); (3) Fluorquinolones class (ciprofloxacin); (4) Tetracycline class (tetracycline, doxycycline); (5) Folate Pathway Inhibitors (co-trimoxazole o trimethoprim/sulfamethoxazole); (6) Phenicols class (chloramphenicol); (7) Penicillin class (oxacillin, ampicillin, amoxicillin, amoxicillin clavulanic acid, penicillin); (8) Glycopeptides class (vancomycin); (9) Macrolides class (erythromycin); (10) Lincosamides class (clindamycin); (11) Fosfomycin class (fosfomycin); (12) and Rifampicin class (rifampicin). The microorganisms cefoxitin resistant were also considered as resistant to methicillin.

### Data analysis

Data were analyzed for descriptive statistics using SPSS version 21 and Microsoft Excel and presented in tables. The results were interpreted in terms of frequencies and percentages.

### Ethical considerations

Ethics committee approvals were obtained from both the local Research and Publication Committee of the GGH and the Health Unit and Ethical Review Committee of the Ethiopian Catholic Secretary. We ensured that the study protocol conformed to the ethical guidelines of the 1975 Declaration of Helsinki as reflected in the approval by the institution’s human research review committee. We also made sure that either oral or written informed consent was obtained from each patient.

## Results and discussion

Forty-four (64.7%) of the 68 patients were male and twenty four (35.3%) were female. The average age of the patients was 43.9 (range age: 18 to 75 years old), and the majority of the patients (n = 38; 55.8%) were aged between 30–50 years old. Clinical data of leprosy patients with ulcers are shown in Table 1. Main localization was the feet (86.8%) (Table 1).

**Table 1:** **Clinical data of 68 leprosy patients with ulcers studied**

	**Number**	**Percentages**
Ulcer condition		
New	7	10.3
Recurrent	61	89.7
Years of leprosy diagnosis		
≤ 1 year	7	10.3
> 1 year to 6 year	15	22.1
> 6 year	46	67.6
Time with ulcer		
< 1 year	53	77.9
≥ 1 year	15	22.1
Ulcer location		
Feet	59	86.8
Legs	6	8.8
Upper extremities	3	4.4
Foot ulcer site [n = 59]		
Sole	41	60.3
Fingers	9	13.2
Heel	5	7.4
Finger and sole	2	2.9
Sole and heel	2	2.9
Osteomyelitis		
Yes	31	45.6
No	37	54.4
White blood cell [x 10^6/^/l] [n = 58]		
< 4.000	4	6.9
4.000 – 11.000	40	69.0
> 11,0	14	24.1
Hemoglobin [g/dl] [n = 55]		
< 12	14	25.5
> 12	41	74.5

SD: standard deviation.

Two (2.9%) out of the 68 cultures performed were negative and 66 were positive. Eighty-one microorganisms were isolated from infected ulcers as shown in Table 2. The main isolation was *Proteus* spp (30.9%), followed by *Escherichia coli* (21.0%), *Staphylococcus aureus*(18.5%) and *Pseudomonas aeruginosa* (9.9%). Gram-positive and Gram-negative bacteria accounted for 24.6% and% 70.5% respectively. Funghal microorganism accounted for 4.9%. Multiple organisms (at least two) were isolated from 15 (22.7% of positive culture) patients. The pattern of mixed growth is shown in Table 2: 13.3% of isolation of *Candida* sp., 26.7% of *P. aeruginosa*, 33.3% of *S. aureus*, 26.7% of *Proteus* spp. and 20.0% of *E. coli*. The isolation of a specific bacteria did not have any association with the development of osteomyelitis.

**Table 2:** **Bacteriological isolates of leprosy ulcers among patients**

**Pathogens**	**Number**	**Percentages**
**Gram-positive pathogens**	20	24.6
*Staphylococcus aureus*	15	18.5
Methicillin resistant *S. aureus*	3	3.7
Coagulase negative Staphylococci	2	2.5
*Enterococcus faecalis*	1	1.2
*Streptococcus agalactiae*	1	1.2
*Streptococcus pyogenes*	1	1.2
**Gram-negative pathogens**	57	70.5
Enterobacteriaceae	49	60.6
*Proteus* spp.	25	30.9
*Proteus mirabilis*	21	25.9
*Proteus vulgaris*	4	4.9
*Escherichia coli*	17	21.0
*Enterobacter cloacae*	2	2.5
*Klebsiella pneumoniae*	2	2.5
*Morganella morganii*	2	2.5
*Providencia rettgeri*	1	1.2
Gram-negative non fermantive		
*Pseudomonas aeruginosa*	8	9.9
**Fungal pathogens**	4	4.9
*Candida* spp	4	4.9
*Candida albicans*	1	1.2
*Candida no albicans*	3	3.7
**Total**	81	100
**Mixed growth**	15*	22.7
**Pattern**		
*Staphylococcus aureus and Proteus* spp	3 **	20.0
*Staphylococcus aureus and Escherichia coli*	2	13.3
*Proteus* spp *and Pseudomonas aeruginosa*	2	13.3
*Proteus* spp *and Escherichia coli*	2	13.3
*Staphylococcus aureus and Pseudomonas aeruginosa*	1	6.7
*Proteus* spp *and Candida* sp	1	6.7
*Pseudomonas aeruginosa and Candida* sp	1	6.7
*Staphylococcus aureus and Enterobacter cloacae*	1	6.7
*Streptococcus agalactiae and Enterobacter cloacae*	1	6.7
*Pseudomonas aeruginosa and Escherichia coli*	1	6.7
*S. aureus and Providencia rettgeri*	1	6.7

* Percentage of 66 patients with positive results of culture of ulcer.

** Percentage of 15 patients with mixed growth culture.

Susceptibility patterns of Gram-positive and Gram-negative bacteria isolated from leprosy ulcers against antimicrobial agents are shown in Table 3. Three isolates of *S. aureus* were methicillin resistant (20%), as they were to cefoxitin. This isolation was sensitive to ciprofloxacin, co-trimoxazole, vancomicyn, and rifampicin. About 20 to 30% of all *S. aureus* isolated were resistant to amoxicillin/clavulanic acid, fosfomicyn, erythromycin, chloramphenicol, ciprofloxacin, co-trimoxazole and clindamycin.

**Table 3:** **Antimicrobial drugs resistance pattern of bacteria isolated from leprosy ulcers**

**Drugs no [%] resistance to**																		
**Bacteria**	**AMP**	**AMX**	**AMC**	**CRO**	**FOX**	**FOS**	**GM**	**E**	**C**	**TE**	**CIP**	**SXT**	**DO**	**CC**	**VA**	**RI**	**P**	**OX**
*S. aureus* [n = 15]	12 [80]	15 [100]	4 [26.7]	6 [40]	3 [20.0]	3 [20.0]	0 [0]	4 [26.7]	3 [20.0]	2 [13.3]	3 [20.0]	4 [26.7]	0	3 [20]	0	0	12 [80]	3 [20]
*Streptococcus* spp [n = 2]	-	-	-	-	-	-	-	1 [50.0]	-	-	-	1 [50.0]	-	-	-	-	0	-
*E. faecalis*[n = 1]	0 [0]	0 [0]	0 [0]	1 [100]	1 [100]	0 [0]	1 [100]				1 [100]	0 [0]	-	-	0	0	0	1 [100]
*Proteus* spp.[n = 25]	17 [68.0]	13 [52.0]	5 [20.0]	2 [8.0]	3 [12.0]	4 [16.0]	8 [32.0]	12 [48.0]	9 [36.0]	24 [96.0]	8 [32.0]	14 [56.0]	7 [28.0]					
*E. coli* [n = 17]	12 [70.6]	11 [64.7]	1 [5.9]	1 [5.9]	2 [11.8]	3 [17.6]	2 [11.8]	7 [41.2]	6 [35.3]	7 [41.2]	5 [29.4]	10 [58.8]	5 [29.4]					
Others* [n = 7]	7 [100]	6 [85.7]	5 [71.4]	4 [57.1]	5 [71.4]	4 [57.1]	2 [28.6]	6 [85.7]	5 [71.4]	5 [71.4]	1 [14.3]	4 [57.1]	4 [57.1]					
Total Enterobacteriaceas [n = 49]	36 [73.5]	30 [61.2]	11 [22.4]	7 [14.3]	10 [20.44]	11 [22.4]	12 [24.5]	25 [51.0]	20 [40.8]	36 [73.5]	14 [28.6]	28 [57.1]	16 [32.7]					
*P. aeruginosa*[n = 8]	8 [100]	8 [100]	8 [100]	7 [87.5]	6 [75.0]	4 [50.0]	2 [25.0]	8 [100]	4 [50.0]	8 [100]	3 [37.5]	8 [100]	8 [100]					

Key: OX = Oxacillin, FOX = Cefoxitin, AMC: Amoxicillin clavulanic acid, E = Erythromycin, CC = Clindamycin, AMP = Ampicillin, AMX; Amoxicilin; P = Penicillin, TE = Tetracycline, DO = Doxycycline, CRO = Ceftriaxone, SXT = Co-trimoxazole, GN = Gentamycin, VA = Vancomycin, CIP = Ciprofloxacin, C = Chloramphenicol, RI: Rifampicin, FOS: Fosfomicyn. *Klebsiella pneumoniae [n = 2], *Enterobacter cloacae* [n = 2], *Morganella morganiii* [n = 2] and *Providentia rettgeri* [n = 1].

More than 50% of Enterobacteriaceae were resistant to tetracycline (73.5%), ampicillin (73.5%), amoxicillin (61.2%) and co-trimoxazole (57.1%); from 40 to 50% were resistant to erythromycin (51.0%) and chloramphenicol (40.8%); from 20 to 40% were resistant to, doxycycline (32.7%), ciprofloxacin (28.6%), gentamycin (24.5%), fosfomicyn (22.4%), amoxicillin clavulanic acid (22.4%) and cefoxitin (18.4%) ; finally, less than 20% were resistant to ceftriaxone (14.3%). From *P. aeruginosa* isolated 50% were resistant to chloramphenicol, 37.5% to ciprofloxacin and 25% to gentamycin.

In the total amount of the isolated bacteria, the antibiotics with less resistance were gentamicin (18.5%), fosfomycin (22.2%), cefoxitin (24.7%), ceftriaxone (25.9%), ciprofloxacin (25.9%), followed by amoxicillin-clavulanic acid (28.4%), doxycycline (29.6%), chloramphenicol (33.3%), erythromycin (46.9%), co-trimoxazole (50.6%) and tetracycline (56.8%). Most of the bacteria were resistant to both amoxicillin (65.4%) and ampicillin (69.1%).

Among the various complications that occur in leprosy are plantar, palmar and corneal ulcerations [14,19] and once these ulcers develop, secondary bacterial infections usually follow. Bacterial etiologies of these infections have not been studied in depth and published information is scarce in Ethiopia [12-14].

Diversified bacteriological agents have been identified in different studies, and in ours the main pathogens were Enterobacteriaceae, followed by *S. aureus* and *P. aeruginosa*, being *Proteus mirabilis* the most common pathogen isolated*.* In a study of Indian leprosy patients with ulcers, by Kumar et al., the most common isolate was *P. aeruginosa* [6], which is similar to studies reported elsewhere [14]. However, the results in most of the studies are around 10% for *P. aeruginosa* [10,14,20], with *S. aureus* being the major isolate in studies carried out in India, South Africa and Mali [9-11,20,21]. In this paper, *S. aureus* was the second bacteria isolate with a prevalence of 18.5%, which is the most virulent of all Staphylococci encountered. The invasive nature of this organism poses a threat for deeper tissue invasion and a potential risk for bacteremia.

Antimicrobial resistance is increasing, which is a worldwide problem that continues to challenge medical practice [22,23], and has become an important concern for the clinician, patients and the pharmaceutical industries in both the hospital and community environment [23]. 20% of cases were resistant to methicillin, higher prevalence than it has been found in others studies about leprosy ulcers (9%) [14]. Maybe it is related to the empirical treatment with cloxacillin previous to this study. Methicillin resistant *S. aureus*(MRSA) is a relevant problem because of the impossibility to treat with methicillin or oxacillin, representing a deeper dilemma in developing countries. Enterobacteriaceae is more than 50% resistant to co-trimoxazole and 20% resistant to ciprofloxacin. The best antimicrobial choice for treating this bacterium is ceftriaxone followed by amoxicillin clavulanic acid and fosfomycin, because it is less prevalent to be resistant. The isolation of *P. aeruginosa* is a problem, because no appropriate antibiotics, such as ceftazidime or carbapenem, are available in rural areas of low-income countries.

When should antibiotics be used to manage leprosy ulcers? According to Lema et al. [14] the use of antibiotics has two main rules: the first rule is ‘do not use antibiotics as a routine’; the second general rule is ‘do not fail to use appropriate antibiotics when needed’. Ulcers with osteomyelitis, however, need antibiotics to recover the affected area from microorganisms and to cure osteomyelitis. Antibiotic treatment is then empirical because cultures of infected ulcers and the sensitivity of the microorganisms are not available in rural areas. After this pilot study in our hospital, we can empirically choose a better antibiotic when it is not possible to perform cultures. According to the results of our study, aminoglycosides (gentamicin), quinolones (ciprofloxacin), fosfomycin, amoxicillin-clavulanic acid and cephalosporin of second and third generation (ceftriaxone, cefoxitin), suggested as treatment options, show less than 30% resistance.

Moreover, it should be noted the fact that daily ulcer care and shoe adjustments are as important as oral antibiotics in these cases. Several investigators have been working with alternative therapeutic options for the management of lepromatous ulcers by using topical agents such as citric acid [22,24] or phenytoin sodium with Zinc oxide [20,25], when ulcers do not heal. Preventive surgery could accelerate the healing of this kind of ulcer. Other conservative approaches, such as orthopedic interventions involve reduction of bone hyper pressure areas, which then enables ulcer healing [26]. Moreover, various loco-regional flaps have been described for the reconstruction of trophic ulcers, but very large defects, on the other hand, are not amenable to local flaps [27]. Other authors used a free tissue transfer form (a radial artery forearm free flap), one of the options for trophic ulcer complicating leprosy [28].

In a systematic review of the literature about the quality of reporting andmethodology of studies on interventions for trophic ulcers in leprosy, Forsetlund and Reinar [29] concluded that the existing infrastructure in the leprosy field, and the presumably restricted funds for treatment and research, may limit the opportunities for undertaking high quality randomized controlled trials. Moreover, the most important threat in existing studies is the threat of selection bias; for instance, there is an apparent need to stimulate more research and improve methodological quality, as well as the quality of reporting the trials in leprosy ulcer treatment [30].

In this study the osteomyelitis presented in 45% of the patients, which was higher than in other studies [8,9,13]. This might be due to the fact that GGH is a reference hospital, where the patients come for ulcer treatment , and there is a team of orthopaedic surgery operating this kind of pathology four times a year. Besides that, some microorganisms such as *S. aureus* have a higher risk to cause osteomyelitis, but in our study the isolation of a specific bacteria did not have any association with osteomyelitis.

This pilot study has some limitations because cultures for anaerobic bacteria could not be performed, nor have we followed the patients up undergoing antibiotic treatment to see the outcome of their ulcers.

Ulcers often lead to morbidity and/or poor quality of life of leprosy patients [14,19,21,25]. The situation with regards to leprosy is pathetic, and hampers the restoration of social status to leprosy patients, which in turn contributes to a greater misunderstanding about the disease spreading [21,25]. Bacteriological study of the ulcers of leprosy patients is appropriate to identify the pathogens and sensitivity. The pathogens in this study are different to those causing disease in others countries, being *Proteus spp* the main pathogen involved. For this reason, it is believed that an effort should be made in order to improve the management of chronic ulcers of leprosy patients. This could begin performing bacterial cultures to guide an appropriate antibiotic, studying alternative treatment in the case conventional treatment fails. Finally we would like to emphasize the importance of a proper health education, daily ulcer care and shoe adjustments as systemic therapy and also to prevent the development of new ulcers.
